# Non-human primate papillomavirus E6-mediated p53 degradation reveals ancient evolutionary adaptation of carcinogenic phenotype to host niche

**DOI:** 10.1371/journal.ppat.1010444

**Published:** 2022-03-25

**Authors:** Teng Long, Robert D. Burk, Paul K. S. Chan, Zigui Chen

**Affiliations:** 1 Department of Microbiology, Faculty of Medicine, The Chinese University of Hong Kong, Hong Kong Special Administrative Region, China; 2 Departments of Pediatrics, Microbiology and Immunology, Epidemiology and Population Health, and Obstetrics, Gynecology and Woman’s Health, Albert Einstein College of Medicine, New York city, New York, United States of America; University of North Carolina at Chapel Hill, UNITED STATES

## Abstract

Non-human primates (NHPs) are infected with papillomaviruses (PVs) closely related to their human counterparts, but there are few studies on the carcinogenicity of NHP-PVs. Using an *in vitro* cell co-transfection assay, we systematically screened the biochemical activity of E6 proteins encoded by macaque PVs for their ability to bind and promote degradation of host p53 proteins. A host species barrier exists between HPV16 and MfPV3 with respect to E6-mediated p53 degradation that is reversed when p53 residue 129 is swapped between human and macaque hosts. Systematic investigation found that E6 proteins encoded by most macaque PV types in the high-risk species α12, but not other Alpha-PV clades or Beta-/Gamma-PV genera, can effectively promote monkey p53 degradation. Interestingly, two macaque PV types (MfPV10 and MmPV1) can simultaneously inhibit the expression of human and monkey p53 proteins, revealing complex cross-host interactions between PV oncogenes and host proteomes. Single point-mutant experiments revealed that E6 residue 47 directly interacts with p53 residue 129 for host-specific degradation. These findings suggest an ancient host niche adaptation toward a carcinogenic phenotype in high-risk primate PV ancestors. Following periods of primate host speciation, a loss-of-function mutation model could be responsible for the formation of a host species barrier to E6-mediated p53 degradation between HPVs and NHP-PVs. Our work lays a genetic and functional basis for PV carcinogenicity, which provides important insights into the origin and evolution of specific pathogens in host pathogenesis.

## Author summary

Many critical questions about the genetic basis underlying HPV carcinogenicity and heterogenicity remain and need to be addressed. In this study, we used analytical tools of phylogenetic and systematic biology by expanding the repertoire of macaque PV genomes and *in vivo* experiments to elucidate the origin and adaptive response of a carcinogenic property in primate PVs. Our data revealed that most macaque PVs that are phylogenetically related to high-risk HPVs, rather than other Alpha-PV clades or Beta-/Gamma-PV genera, effectively promote the degradation of host p53, suggesting an evolved phenotype inherited from a common ancestor of high-risk primate PVs. A host species barrier to E6-mediated p53 degradation was commonly observed between HPVs and NHP-PVs. Interestingly, for the first time, we showed that two macaque PV types exhibit a potential for cross-host p53 degradation, implying complex pathogen-host interactions that may not strictly segregate from carcinogenicity and host specificity. The emergence of carcinogenic phenotypes in high-risk primate PVs might be the result of an ancient adaptation of viral ancestors to specific host niches. Overall, this study expands the picture of PV phenotype-genotype relationship from an evolutionary perspective of genetics and function, laying an important foundation for understanding viral fitness and its pathological consequences.

## Introduction

Papillomaviruses (PVs) are a heterogeneous group of epithelium-tropic DNA viruses with genomes that are about 8,000 nucleotides in size and that infect epithelial surfaces of vertebrates ranging from fish to mammals. PVs are highly species-specific and have co-evolved with their hosts for millions of years [1]. To date, the complete genomes of more than 600 distinct PV types have been characterized. Human papillomaviruses (HPVs) are phylogenetically grouped into five genera: Alpha-, Beta-, Gamma-, Mu-, and Nu-PV (https://pave.niaid.nih.gov; accessed on 22 October 2021) [2,3]. Clinical consequences of infection with HPVs range from asymptomatic and benign warts to persistent infection with high-risk Alpha-HPV types, such as HPV types 16 (HPV16) and 18 (HPV18), can lead to the development of high-grade lesions, invasive cervical cancer or cancers of the lower genital tract and head and neck regions [4]. As anticipated by the generality of PV coevolution with hosts, non-human primates are infected with viruses closely related to HPVs. HPVs and non-human primate PVs (NHP-PVs) share a similar evolutionary history and have adapted to variable host niches such as the mucosal and cutaneous ecosystems [5–8]. For example, the carcinogenic HPV types that cause essentially all cervical cancers are phylogenetically clustered in one clade consisting of species α5, 6, 7, 9, and 11 [9]. Interestingly, some mucosal macaque PV types in the species α12, such as *Macaca fascicularis* papillomavirus type 3 (MfPV3) that is associated with cervical cancer, share a most recent common ancestor (MRCA) with that of the human-infecting high-risk HPV clade [10,11], suggesting that primate PV ancestors containing oncogenic determinants may have adapted to specific host niches prior to the speciation of host ancestors.

PV genomes typically contain eight open reading frames (ORFs) that encode six early products (E1, E2, E4, E5, E6, and E7) and two late products (L1 and L2) [12]. E5, E6 and E7 proteins promote the viral life cycle by manipulating the balance of cell proliferation and differentiation to provide an environment conducive both to persistent infection in basal cells and to productive infection in differentiated cells [13]. Oncogenic Alpha-HPVs could disrupt this balance and inactivate cell cycle checkpoints in the less-differentiated lower layers, which facilitates the viral life cycle and leads to a high frequency of genetic instability in the cell and malignant progression [14]. Whereas E5 is not required for carcinogenesis, persistent overexpression of carcinogenic HPV E6 and E7 proteins is able to disrupt important regulatory pathways, most notably those mediated by the retinoblastoma protein family (pRB, p107, and p130) [15], p53 [16], and PDZ domain-containing proteins [17]. As a key regulator of cell proliferation, p53 is important for the induction of cell cycle arrest and apoptosis upon aberrant cell cycle progression [18]. Both carcinogenic and non-carcinogenic HPV E6 proteins form a complex with E3 ubiquitin ligase E6-associated protein (E6AP) to inactivate p53 transactivation resulting from unscheduled E7-mediated DNA synthesis. However, only carcinogenic HPV E6 proteins stimulate polyubiquitination of p53 and subsequent proteosome-dependent degradation to induce keratinocyte immortalization [16,19]. Although HPV E6s can localize to the nucleus and cytoplasmic membrane [20], carcinogenic HPV E6s are co-exported with p53 from the nucleus to the cytoplasm to promote p53 degradation [21]. In contrast, E6 proteins encoded by non-carcinogenic HPVs may trap p53 in the cytoplasm and induce apoptosis [22]. Cooperative actions of the carcinogenic E6 and E7 proteins abrogate multiple cell cycle checkpoints and critical pathways that are essential for maintaining cellular homeostasis [23–25]. As a consequence of these activities, in the absence of p53, cells do not undergo growth arrest upon E6/E7-induced DNA damage, thereby increasing the frequency of genome instability towards malignancy. Synergistically, the carcinogenic E6 and E7 proteins evade host immunity, with a consequence of increasing the susceptibility of cells to oncogenesis [26].

It is not clear what genetic dynamics are responsible for the large variability in the risk of cancer conferred by different PV types. In view of an evolutionary conservation between HPVs and NHP-PVs related to tissue tropism and carcinogenicity, determining their genetic and functional similarities may contribute to a better understanding of the origin and adaptive response of carcinogenic PV to host ecosystems. In this study, we applied an *in vitro* co-transfection assay to determine the ability of NHP-PV E6s to bind and promote host p53 degradation. Macaque PV types belonging to Alpha- (high- and low-risk clades), Beta- and Gamma-PV genera were included, which allowed us to evaluate this phenotype from an evolutionary perspective. Key sequence features in E6 and p53 proteins critical for host-specific degradation were identified that may represent a novel therapeutic target for HPV-induced cervical cancer. Our findings implicate an ancient niche adaptation of a carcinogenic phenotype and provide important insights into the complex interactions between viral pathogens and host proteomes in cancer pathogenesis.

## Results

### Phylogeny of primate papillomaviruses

**Fig 1:**
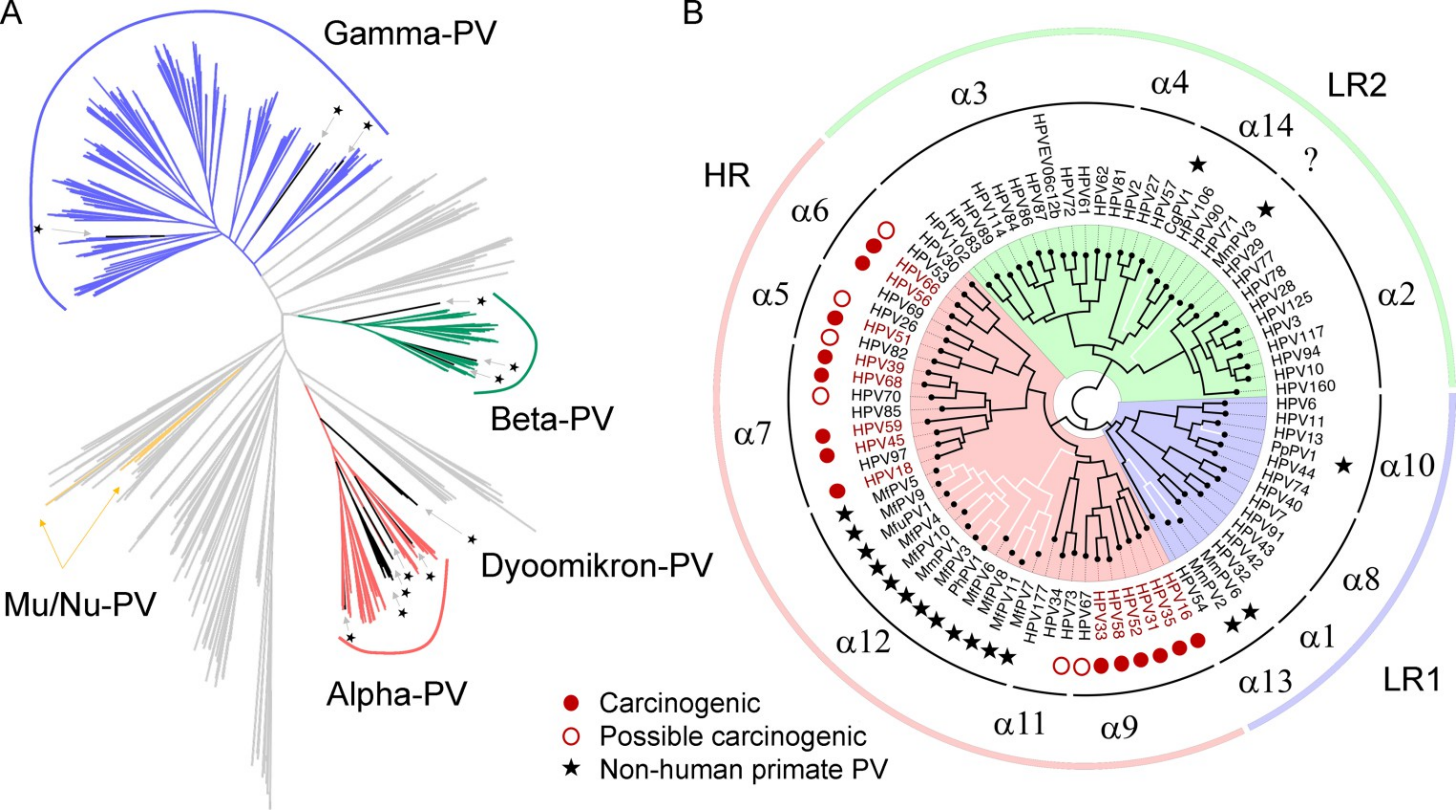
Phylogeny of papillomaviruses. **(A)** Maximum likelihood tree inferred from concatenated nucleotide sequences of four open reading frames (ORFs) (E1, E2, L2, and L1) of 640 papillomavirus genomes including 442 HPVs, 27 non-human primate PVs (NHP-PVs) and 171 non-primate animal PVs (NPA-PVs). All characterized primate PVs (HPVs and NHP-PVs) clustered into Alpha-, Beta-, Gamma-, Mu-, Nu-, and Dyoomikron-PV genera. Asterisks indicate branches/clades of non-human primate PVs. NPA-PVs are represented by gray lines. Accession numbers of the PV genomes were listed in S1 Table. **(B)** Maximum likelihood tree of the Alpha-PV genus inferred from concatenated nucleotide sequences of six ORFs (E6, E7, E1, E2, L2, and L1) of 66 HPV types and 17 NHP-PV types. At least three ancestral PVs are responsible for the current heterogeneous groups of genital PVs including high-risk (HR, α5/6/7/9/11/12), low-risk 1 (LR1, α1/8/10/13) and LR2 (α2/3/4/14). NHP-PV genomes are joined by white lines. Carcinogenic and possibly carcinogenic HPV types defined by the International Agency for Research on Cancer (IARC) are indicated by red dots and open circles, respectively. NHP-PV types are indicated by asterisks. Question mark indicates unknown classification.

To better understand the evolutionary history of primate papillomaviruses, we first assessed the phylogeny of the family *Papillomaviridae* inferred from concatenated nucleotide sequences of the four largest open reading frames (ORFs) (E1, E2, L2, and L1) of 640 PV complete genomes (S1 Data). NHP-PVs shared a common ancestry with human counterparts (Fig 1A). All characterized primate PVs were clustered into the genera Alpha-, Beta-, Gamma-, Mu-, Nu- and Dyoomikron-PV, with Alpha/Dyoomikron-PVs being relatively distant from the Beta/Gamma-PVs. At least three mucosal primate PV ancestors, including high-risk (HR, α5/6/7/9/11/12), low-risk 1 (LR1, α1/8/10/13) and LR2 (α2/3/4/14), were responsible for the current heterogeneity of Alpha-PVs (Fig 1B). In the high-risk Alpha-PV clade, a large set of NHP-PVs in the species α12, each isolated from the cervicovaginal region of macaques, shared a most recent common ancestor (MRCA) with the genital high-risk HPV types (e.g., HPV16 within the species α9).

### Subcellular co-localization of PV E6 and host p53 proteins

**Fig 2:**
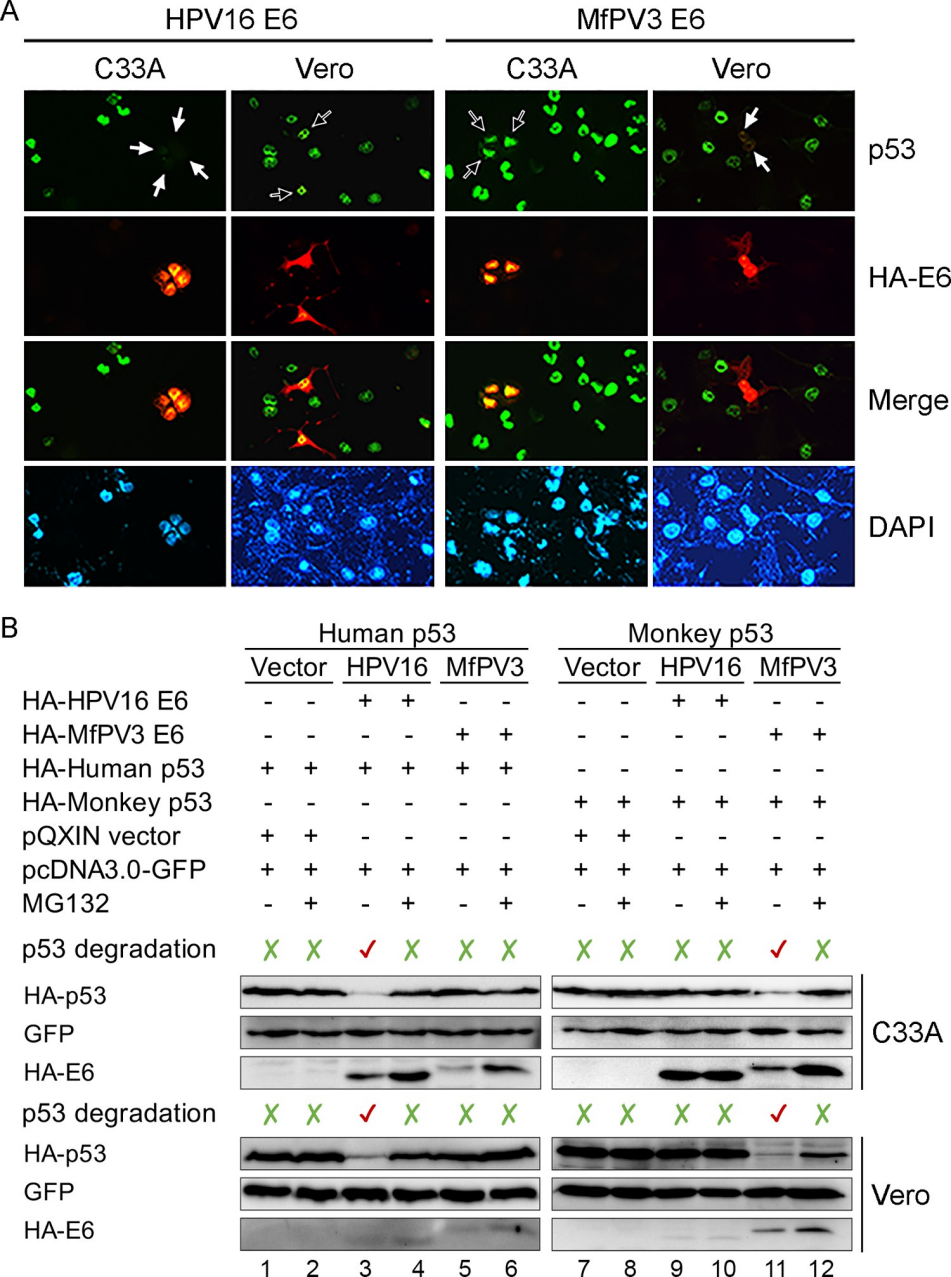
Primate PV E6-mediated p53 degradation. **(A)** Double immunofluorescence assay to localize the expression of E6 and p53 proteins in C33A (human) and Vero (green monkey) cells. Endogenous p53 and HA-tagged E6 proteins were stained green and red, respectively. Blank arrows indicate the location where E6 and p53 were co-expressed in cells, whereas white arrows denote E6-mediated p53 degradation. Nuclei of cells were counterstained with DAPI. Images were taken by an All-in-One fluorescence microscope with 400X magnification (Leica DM1000, Wetzlar, Germany). **(B)** Host-specific p53 degradation mediated by E6 proteins. HA-tagged p53 (human *vs* monkey) and E6 (HPV16 *vs* MfPV3) were co-transfected *in vitro* in C33A or Vero cells, respectively. Lanes 1, 3, 5, 7, 9, and 11 show results from co-transfected HA-p53 and vectors; lanes 2, 4, 6, 8, 10, and 12 show the p53 expression levels after treatment with MG132. The expression levels of green fluorescent protein (GFP) are also shown as a co-transfection control.

Two carcinogenic primate PV types, HPV16 and MfPV3 representing the high-risk species α9 and α12, respectively, were selected to localize the subcellular expression of E6 and p53 proteins in the cell. As shown in Fig 2A, E6 proteins encoded by both HPV16 and MfPV3 were well-expressed in the nucleus of cells. Interestingly, a host species barrier to E6-mediated p53 degradation was observed: MfPV3 E6 effectively reduced the expression level of monkey p53 protein in Vero cells but had almost no activity on human p53 degradation in C33A cells. Similarly, HPV16 E6-mediated p53 degradation was only observed in C33A cells (Fig 2A).

### *In vitro* assessment of host species barrier to E6-induced p53 degradation

To assess the ability of MfPV3 E6 to promote p53 degradation, an *in vitro* co-transfection assay by overexpressing the E6 and p53 proteins in C33A or in Vero cell lines was applied. Consistent with the subcellular co-localization results, the expression levels of HA-tagged monkey p53 proteins were dramatically reduced when co-transfected with the E6 gene of MfPV3, but not HPV16, and vice versa (Fig 2B). In addition, treatment with the proteasome inhibitor MG132 restored the HA-p53 levels in cells expressing E6 proteins, confirming that primate PV E6-mediated p53 degradation is a ubiquitin proteasome-dependent event.

### Structure-based alignment of human and macaque p53 proteins

**Fig 3:**
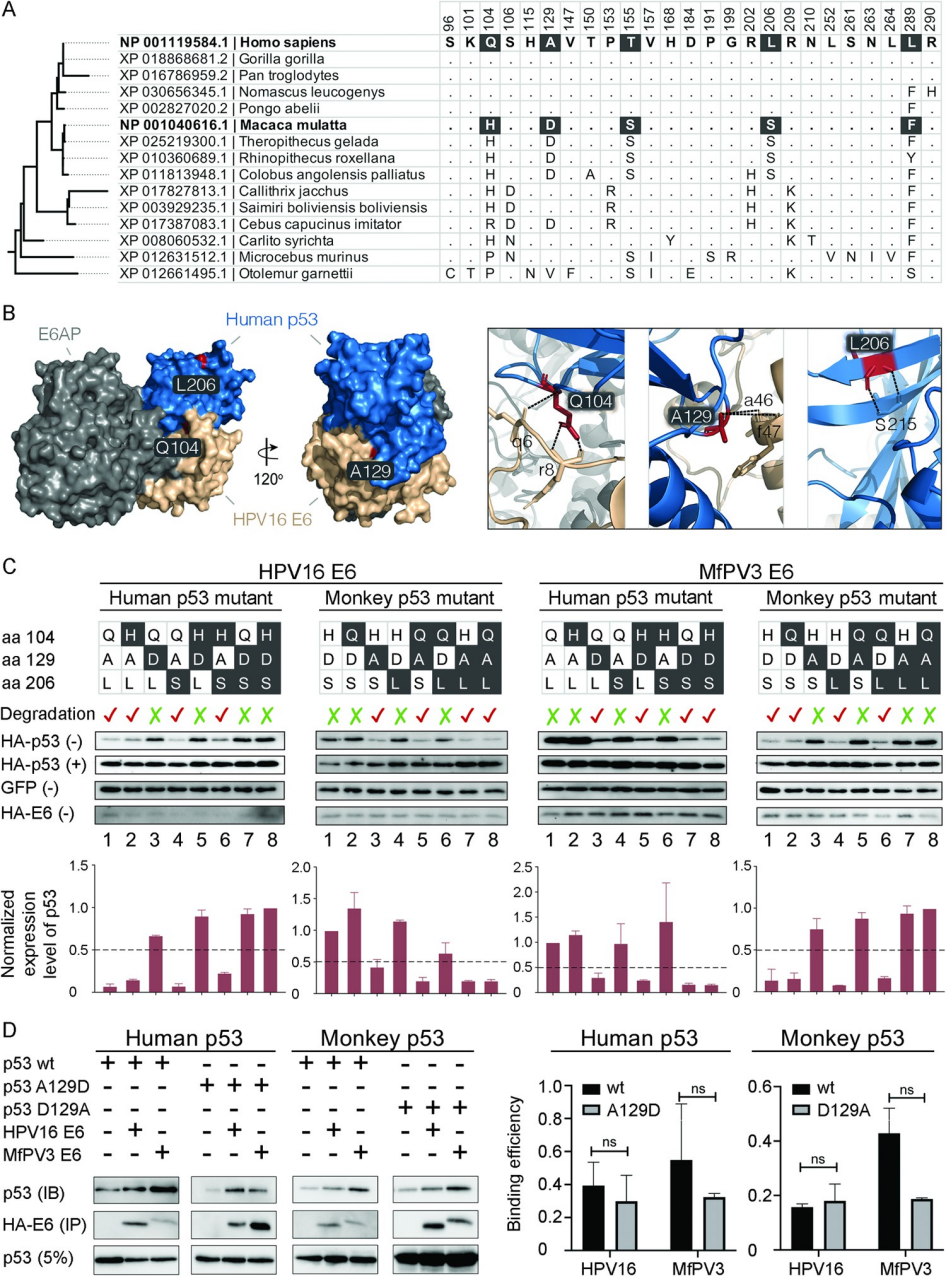
p53 residue 129 for host species-specific E6-mediated degradation. **(A)** Amino acid alignment of the E6-binding domain (aa 94–292) of p53 in major primate hosts identified five distinguishing residues (104Q/H, 129A/D, 155T/S, 206L/S, and 289L/F) between *Homo sapiens* and *Macaca mulatta*. **(B)** Estimated structures of the HPV16 E6, human p53 and E6AP complexes. Surface representation of the p53, E6 and E6AP domains are colored in blue, tan and grey, respectively. Three indicated p53 residues (Gln104, Ala129 and Leu206) are denoted in red. Views of sub-interfaces of these three mutations are shown in the boxes on the right panel of the figure. Direct polar interactions are presented with dashed lines. Residues of p53 and E6 are indicated in upper- and lower-case, respectively. **(C)** Degradation of human and monkey p53 mutants mediated by HPV16 and MfPV3 E6s in C33A cells. Bar charts below the western blot images show the normalized expression levels of p53 against GFP. (+) and (-) indicates treatment with and without proteasome inhibitor MG132, respectively. The expression levels of GFP and HA-tagged E6 treated with MG132 are shown in S2 and S3 Figs. **(D)** Co-immunoprecipitation of HA-E6 and HA-p53 in H1299 cells indicates that residue 129 of the p53 mutants appeared to have no impact on the binding activity to the E6/p53 complex compared to the wild-type. IB: immunoblotting IP: immunoprecipitation; 5%: 5% loading of supernatant before IP. The bar charts on the right panel of the figure show the mean (± SEM) values of the binding efficiency inferred in triplicate samples. No statistical significance (ns, Student’s *t-*test) was observed between the wild-type and mutant p53.

We assessed p53 amino acid homology in major primate hosts to identify mutations associated with host-specific p53 degradation potential (Fig 3A). Five amino acid changes (aa 104, 129, 155, 206, and 289) within the E6-binding core domain (aa 94–297) of p53 were observed between humans and macaques; of these, 104Q/H (Glutamine ↔ Histidine), 129A/D (Alanine ↔ Aspartic acid) and 206L/S (Leucine ↔ Serine) showed differential atomic properties to each other (S1 Table). In particular, by simulating the crystal structure of E6-E6AP-p53 binding, it was found that p53 residues 104 and 129 were located in the interfacial areas of the E6/p53 complex (Figs 3B and S1). Besides, humans and macaques shared identical E6AP core proteins.

### p53 residue 129 is important for E6-mediated degradation

Site-directed mutagenesis was performed to swap the p53 amino acid changes at residues 104, 129 and 206 homologous to humans and macaques, resulting in fourteen p53 mutants (human p53: Q104H, A129D, L206S, Q104H/A129D, Q104H/L206S, A129D/L206S, and Q104H/A129D/L206S; monkey p53: H104Q, D129A, S206L, H104Q/D129A, H104Q/S206L, D129A/S206L, and H104Q/D129A/S206L). We then co-transfected p53 mutants and E6 genes *in vitro* using C33A and Vero cells to compare the differential expression of p53 proteins (Figs 3C and S2 and S3). Compared to the wild-type (wt) p53 protein, HPV16 E6-mediated degradation was abolished when the E6 genes were co-transfected with human p53 A129D or mutants containing the A129D change (Q104H/A129D, A129D/L206S and Q104H/A129D/L206S) (Fig 3C, lanes 3, 5, 7 and 8 of the first left panel). Similar degradation patterns of the monkey p53 mutants mediated by MfPV3 E6 were also observed (Fig 3C, the fourth panel). In contrast, mutants of residues 104 and 206, alone or in combination, did not diminish the activity of E6 proteins on p53 degradation. Interestingly, the host species barrier to E6-mediated p53 degradation was overcome when p53 residue 129 was swapped between humans and macaques. For example, MfPV3 E6 well degraded human p53 A129D; similarly, the expression of monkey p53 mutants containing the D129A change was significantly inhibited by HPV16 E6 (Fig 3C, lanes of 3, 5, 7 and 8 of the second and third panels). Notably, p53 mutants at residue 129 appeared to have no impact on the binding activity to the E6/E6AP/p53 complex compared to the wild-type proteins (Figs 3D and S4). Taken together, p53 residue 129 plays an important role in interfacing the E6/E6AP/p53 complex for E6-mediated degradation, which is highly host species-specific.

### Evolutionary relatedness of non-human primate PV E6-mediated p53 degradation

**Fig 4:**
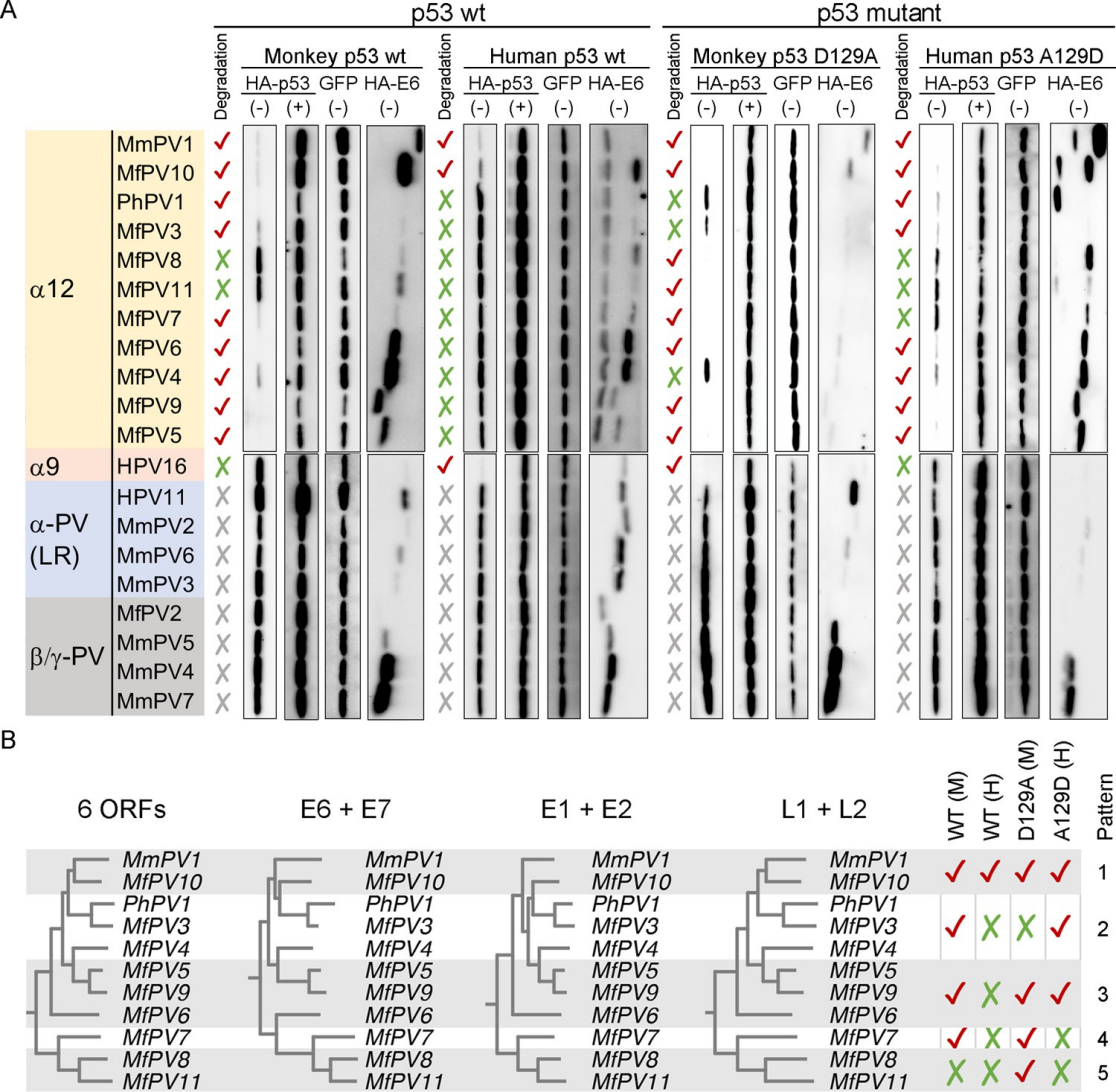
Evolutionary relatedness of non-human primate PV E6-mediated p53 degradation. **(A)** Degradation of p53 proteins mediated by macaque PV E6s encoded by major NHP-PV species and genera. C33A and Vero cells were used for co-transfection of human and monkey p53 plasmids, respectively. (+) and (-) at the top of the images indicate treatment with and without MG132, respectively. The expression levels of GFP and HA-tagged E6 treated with MG132 are shown in S5 Fig. **(B)** Five patterns of primate PV E6-mediated p53 degradation in the species α12. Phylogenetic trees were constructed using RAxML based on concatenated nucleotide sequences of ORFs.

It has been suggested that the biochemical activity of HPV E6 proteins is an evolved phenotype which needs to be interpreted in the context of evolution [27–29]. However, the evolutionary relevance of NHP-PV E6s mediating host p53 degradation is unknown. In this study, macaque PV E6s encoded by major NHP-PV species and genera were overexpressed *in vitro* to compare their abilities to promote host p53 degradation (Figs 4A and S5). A total of 11 NHP-PV types in the species α12 (MfPV3, 4, 5, 6, 7, 8, 9, 10, 11, MmPV1, and PhPV1), three types in the low-risk clade of Alpha-PV (MmPV2, 3, and 6), and four types in the genera Beta-/Gamma-PV (MfPV2, MmPV4, 5, and 7) were included in this study. As expected, macaque PV E6s belonging to the low-risk clade of genital Alpha-PV and the cutaneous Beta/Gamma-PV did not show any activity to degrade any form of the p53 proteins, whereas most types in the species α12, with the exception of MfPV8 and 11, effectively inhibited the expression of the wild-type monkey p53 protein (Fig 4A). Interestingly, overexpression of MfPV10 and MmPV1 E6s significantly reduced the expression levels of the wild-type p53 proteins across humans and macaques, suggesting that some macaque PV types acquire the potential for cross-host p53 degradation. When p53 residue 129 was swapped between humans and macaques (Alanine ↔ Aspartic acid), our *in vitro* co-transfection assay revealed highly complex pathogen-host interactions, with five patterns of E6-mediated p53 degradation in the species α12 (Fig 4A and 4B). Pattern 1 had the potential to degrade human and monkey p53 proteins across hosts, whereas pattern 5 failed to inhibit the expression of the wild-type p53 but instead promoted the degradation of monkey p53 D129A. Although patterns 2, 3 and 4 exhibited a strict host species barrier to wild-type p53 degradation, their abilities to overcome this bottleneck varied and may undergo complex evolutionary histories (Fig 4B).

### Identification of E6 amino acid residues interacting with p53

**Fig 5:**
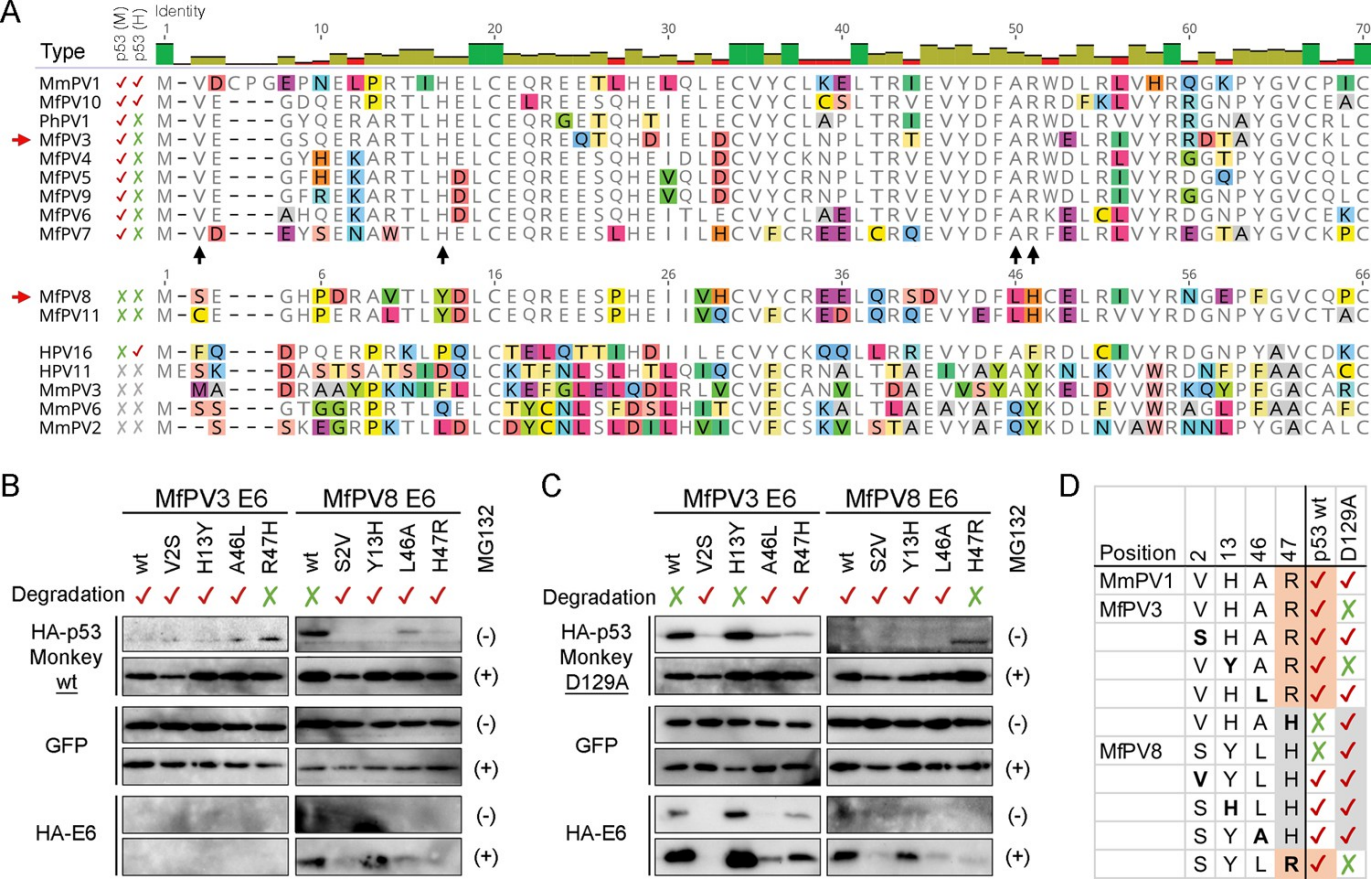
Macaque PV E6 mutagenesis and degradation of p53. **(A)** N-terminal amino acid alignment of macaque Alpha-PV E6s. Activities of the E6s to promote degradation of the wild-type monkey (M) and human (H) p53 are also indicated. Black arrows indicate columns containing homologous amino acids in α12 degraders (Val2, His13, and Arg47) and a residue (Ala46) critical for the E6/p53 interface, whereas red arrows indicate two representative MfPV types for E6 metagenesis. **(B)** Degradation of the wild-type monkey p53 mediated by MfPV E6 mutants. **(C)** Degradation of monkey p53 D129A mediated by MfPV E6 mutants. HA-E6 and HA-p53 were co-transfected in Vero cells. (+) and (-) on the right side of the images indicate treatment with and without MG132, respectively. **(D)** p53 degradation patterns mediated by E6 mutants reveal an important interaction between E6 residue 47 and p53 residue 129.

With the notion that the species α12 shares ancient traits of MRCA with high-risk Alpha-HPVs (Fig 1B), MfPV8 and MfPV11 E6s should contain mutation(s) defective for p53 degradation. To test this hypothesis, we compared the amino acid sequences at the N-terminus of NHP-PV E6s that interface with the E6/E6AP/p53 complex and found three homologous residues in monkey p53 degraders compared to MfPV8/11 and other low-risk NHP-PVs (MfPV3 *vs* MfPV8, E6 aa sites Val2, His13, and Arg47) (Fig 5A, see black arrows). Furthermore, E6 aa Ala46 has been reported to be a critical residue exposed in the E6/p53 interfacial area (Fig 3B) [16], although some PV E6s with opposite effects on monkey p53 stability also contain a homologues Alanine residue. To investigate the effects of these four E6 residues in mediating p53 degradation, we constructed single-point mutant E6 plasmids that were swapped between MfPV3 (V2S, H13Y, A46L, and R47H) and MfPV8 (S2V, Y13H, L46A, and H47R) and co-transfected them with monkey p53 plasmids in Vero cells. Three MfPV3 E6 mutants (V2S, H13Y and A46L), except for R47H, could inhibit the expression of the wild-type monkey p53 as effectively as the prototype E6 (Fig 5B). In contrast, all four single-point mutants of MfPV8 E6s resumed the ability to promote the degradation of the wild-type monkey p53 protein. When the E6 mutants were co-transfected with monkey p53 D129A (Fig 5C), E6s containing His47 likely interacted with p53 Ala129 for degradation (Fig 5D). Meanwhile, E6 residue Arg47 may play an important role in interacting with p53 residue Asp129.

### Loss-of-function mutation of high-risk primate PV E6 in mediating p53 degradation

**Fig 6:**
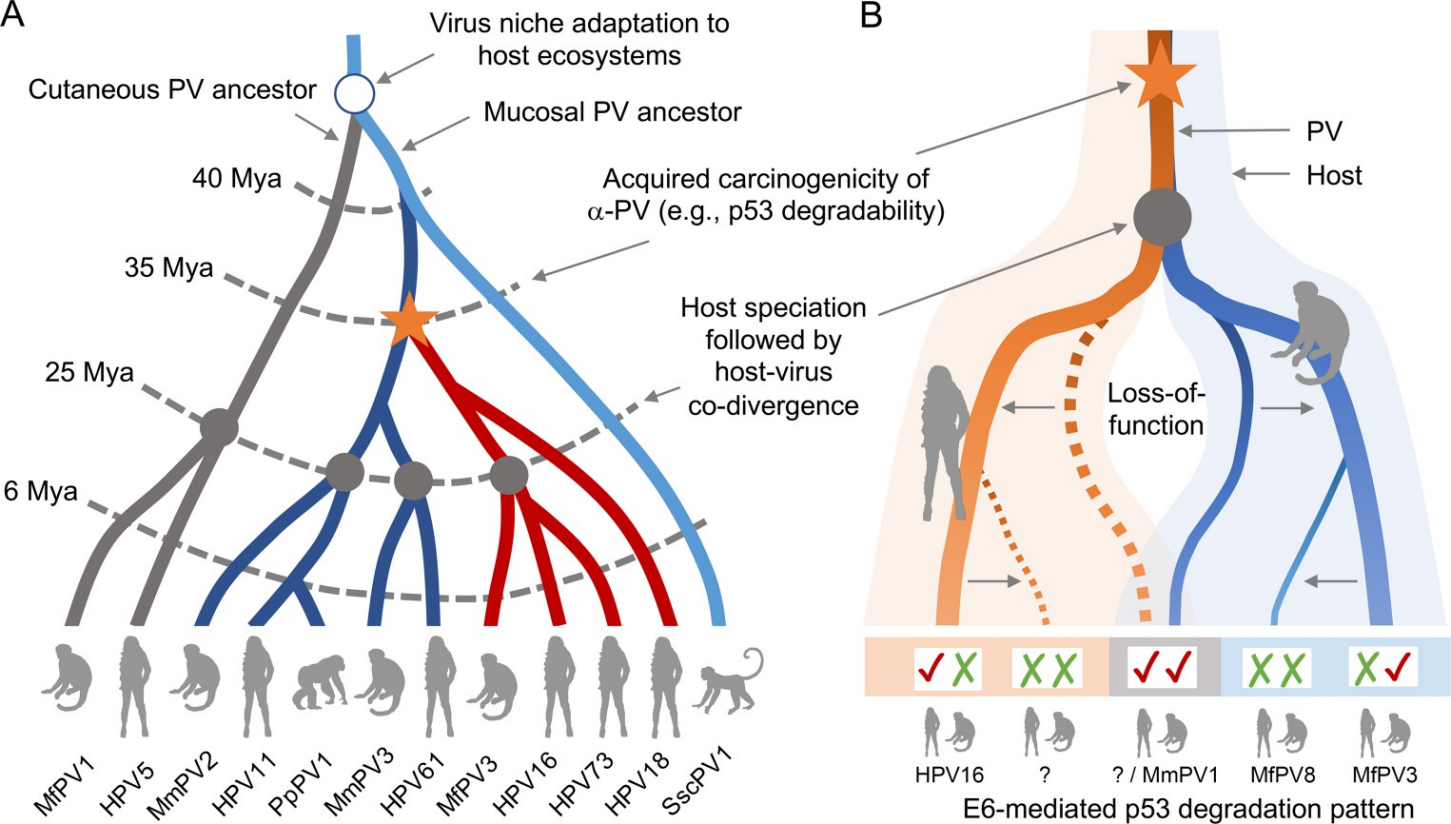
Schematic diagram of a loss-of-function mutation model for the differential abilities of high-risk primate PV E6s to promote p53 degradation. **(A)** Ancient niche adaptation of primate PV ancestors to different host ecosystems (e.g., mucosal *vs* cutaneous) followed by the emergence of carcinogenic PVs (branches in red) and subsequent host speciation events. Divergence times (million years ago, Mya) refer to previous publications [7,8]. Representative PV types are indicated at the end of branches. **(B)** Function loss of acquired viral phenotype following host speciation and viral niche adaptation. This loss-of-function mutation model may be responsible for the formation of a host species barrier or even defective phenotype. Broken lines represent clades in which specific monkey PVs lack detectable human counterparts. Patterns of high-risk primate PV E6-mediated p53 degradation are shown at the bottom of the figure. “√√” indicates cross-host p53 degradation mediated by primate PV E6; “√X” indicates host-specific human p53 degradation mediated by HPV E6; “X√” indicates host-specific monkey p53 degradation mediated macaque PV E6; and “XX” indicates defective p53 degradation mediated by PV E6.

*In vitro* co-transfection of primate PV E6s and host p53 proteins has revealed complex pathogen-host interactions. Our previous work and other studies have demonstrated an ancient niche adaptation of PV ancestors to different host ecosystems (e.g., mucosal *vs* cutaneous) as the first stage of the emergence of carcinogenic PVs (Fig 6A, red lines) [5–8]. This model is consistent with our observation that the most recent common ancestor of the high-risk clade of Alpha-PV has likely acquired phenotypic traits of carcinogenicity (e.g., p53 degradability) before the speciation of primate hosts (Fig 6B), which explains that some macaque PVs, such as MfPV10 and MmPV1, could promote p53 degradation across humans and macaques. Following subsequent host-virus co-divergence, however, the function loss of cross-host p53 degradability likely occurred in most high-risk primate PVs as they adapted to specific host niches, which is consistent with the finding that most α12 types only inhibit expressions of monkey p53. This loss-of-function mutation model may be responsible for the formation of a host species barrier or even defective phenotypic traits, such as the inability of MfPV8 and MfPV11 to promote p53 degradation.

## Discussion

In this work, we applied an *in vitro* co-transfection assay to assess PV E6-mediated degradation of host p53 by expanding the repertoire of non-human primate PVs. Our data revealed that most macaque PVs that are phylogenetically related to high-risk HPVs, rather than other Alpha-PV clades or Beta-/Gamma-PV genera, could effectively promote the degradation of monkey p53. It suggests that the potential for p53 degradation is an evolved phenotype resulting from an ancient host niche adaptation of high-risk primate PVs. Interestingly, for the first time, we found that two macaque PV types in the species α12 could simultaneously inhibit the expression of human and monkey p53 proteins, revealing complex interactions between PV oncogenes and host proteomes. The ability to promote cross-host p53 degradation might be an acquired trait in a most recent common ancestor of high-risk primate PVs prior to the speciation of host ancestors. Following subsequent events of host-virus co-divergence, a function loss seems to be the most phylogenetically consistent explanation of the fact that most PV types in the high-risk Alpha-PV clade exhibited the potential for p53 degradation only in the same host. This loss-of-function mutation model could also be responsible for the reduced biochemical activity of some viruses, such as defective p53 degradation in some α12 NHP-PV types or attenuated oncogenicity in some α5/α6 HPV types. For cross-host p53 degradation, however, the possibility of host interbreeding or independent acquisition of enhanced biological traits should not be ruled out if high-risk HPV ancestors could establish persistent infection in non-human primate ancestors in the early stage of host speciation, and vice versa.

Oncoprotein E6-mediated p53 degradation is thought to be an essential but not self-sufficient activity by which high-risk HPVs contribute to cancer development. For example, HPV30 in α6 and HPV34 in α11 that could decrease p53 levels are not classified as the carcinogenic types [27]. Similarly, most NHP-PVs in the species α12 have inherited the p53 degradation phenotype irrespective of their carcinogenic potential [10,11]. However, the ability to promote p53 degradation could allow the PV ancestors to establish successful colonization in specific host niches. Additionally acquired biochemical activities, such as PDZ degradation [29,30], innate immune evasion [31–33] or other mechanisms utilized by the E6 or E7 oncogenes [12,13,15], may further distinguish closely related carcinogenic viruses from non-carcinogenic members of the high-risk Alpha-PV clade. For example, a PDZ-binding motif (ASRV) present in the C-terminus of MmPV1 E7 but not E6 could direct the interaction with the cell polarity regulator Par3 [34]. Hence, identifying additional phenotypes that regulate host tumorigenesis by epidemiologically carcinogenic types will lead to a better understanding of the evolutionary relationships between PV oncogenes and host proteomes.

Tumor suppressor protein p53 restricts cell growth when cells re-enter cell cycle or are damaged. A high mutation frequency of p53 is observed in nearly all non-HPV cancers, except in cervical tumors, in which p53 is almost invariably the wild-type, indicating that the inhibitory effect of E6 is analogous to an inactivating mutant [35]. This has led to the notion that restoring the activity of p53 may be functional in cervical cancer. For example, functional reactivation of p53 by blocking the ubiquitin-proteasome system can sensitize HPV16-immortalized keratinocytes to CD95-induced apoptosis [36]. Similarly, p53 R175H was reported to inhibit HPV16/18 E6-mediated p53 degradation and restore the pro-apoptotic effects of p53, suggesting a new possibility of designing therapy for cancer patients by p53 reactivation [37]. In this study, we found that a swapped human p53 A129D mutant is defective for HPV16 E6-mediated degradation, which restores its expression level in a proteosome-dependent manner by forming a complex with E6/E6AP. Furthermore, consistent with the reported dominant-negative phenotype of the HPV16 E6 F47R mutant in mediating p53 degradation [38], mutations disrupting the Arg47 (MfPV3 E6 R47H)–Asp129 (monkey p53 D129A) or Phe47 (HPV16 E6 F47R)–Ala129 (human p53 A129D) interactions dramatically impaired p53 degradation. Taken together, these findings suggest that therapeutic strategies targeting the E6-p53 interaction to reactivate the function of p53 would be feasible if the physical association between E6 and p53 could be disrupted [39,40].

To our knowledge, the human p53 A129D mutant does not occur naturally in the general population [41]. Besides, we did not observe an HPV16 E6 variant encoding arginine at position 47 (F47R) [7,42], suggesting the need for a promising approach to permanently inactivate E6–p53 interactions by targeted mutagenesis. The CRISPR/Cas9 system, for instance, has emerged as a powerful genome editing tool to introduce mutations in the carcinogenic E6 and E7 genes to disrupt their functions, thereby inhibiting cell growth and inducing apoptosis in cervical cancer cell lines, which may show great potential in clinical treatment for cervical precancerous lesions [43–45]. HPV oncoproteins dysregulate many regulatory pathways to promote each stage of infection in the cell during their long evolutionary association with their host [29–31,46]. Since most studies on PV biochemical activities have focused on a few high-risk HPV types, such as HPV16 and HPV18, understanding the genetic and functional associations with other members of HPV and NHP-PV would allow us to develop better strategies for patient management (e.g., prophylactic vaccination, screening, and immune modulation) and disease treatment (e.g., targeted antiviral or immunotherapy drugs, and gene editing). A systematic analysis of viral-host interactions combining genetics, biochemistry, evolution, and epidemiology will shed important light on the relative role of specific pathogen phenotypes in host pathogenesis.

One limitation of this study is the lack of a systematic characterization of p53 degradation by Alpha-HPV E6s in NHP hosts. The apoptotic effects of human p53 A129D blocking E6-mediated degradation also warrants further studies *in vitro* and *in vivo*. Another limitation is that we only targeted the E6-binding domain of the p53 gene while other regions may also be involved in the regulation of HPV-associated malignancies. Nevertheless, PV genomes represent an ancient model system for identifying key cellular elements in cancer development. A systematic analysis of NHP-PV E6-mediated p53 degradation in light of evolution provides genetic and functional evidence for understanding the origin and adaptive response of specific carcinogenic phenotypes in host niches.

In summary, we used analytical tools of evolution and systematic biology by expanding the repertoire of macaque PV genomes and *in vivo* experiments to elucidate the potential of E6 for p53 degradation, one of the most important cellular pathways inducing HPV-associated cancer. The acquisition of proteosome-dependent degradation by high-risk primate PV ancestors is an evolved phenotype that allows viruses to establish successful colonization in specific cellular niches. This phenotype probably does not strictly segregate from carcinogenicity and host specificity but a loss-of-function mutation model could be responsible for the formation of a host species barrier and viral fitness to support viral replication in various ways. Understanding the evolutionary relatedness and molecular basis underlying the mechanism of specific pathogens in host pathogenesis will be important in designing novel strategies for patient management and disease treatment.

## Materials and methods

### Ethics statement

This study was approved by the Agriculture, Fisheries and Conservation Department of the Hong Kong SAR Government and The Chinese University of Hong Kong–New Territories East Cluster Clinical Research Ethics Committee (CREC 2016.186). All experiments were performed in accordance with the relevant guidelines and regulations.

### Phylogenetic tree construction

Nucleotide sequences were translation aligned based on aligned amino acid sequences using MUSCLE v3.8.42 [47] in Geneious Prime 2021.0.1 (http://www.geneious.com/). Maximum likelihood (ML) trees inferred from nucleotide sequence alignment of distinct or concatenated ORF(s) were constructed using RAxML MPI v8.2.3 [48] with optimized parameters. Data were bootstrap resampled 1,000 times. The CIPRES Science Gateway [49] was accessed to facilitate RAxML high-performance computation. Phylogenetic trees were visualized using FigTree v1.4.4 (https://github.com/rambaut/figtree/releases).

### Plasmids and transfection

E6 ORFs were PCR-amplified from previously genotyped specimens or genomes using a 5’ primer that included a hemagglutinin (HA) tag sequence in frame with the gene and cloned into the *Not*I*-Eco*RI sites of the pQCXIN vector (Invitrogen, CA, USA) [8,11,50–52]. Human and monkey p53 ORFs, incorporated with an HA-tag at the amino-terminus or three FLAG-tags at the carboxy-terminus, were PCR-amplified from a plasmid provided by Dr. Liang Zhu (Albert Einstein College of Medicine) (NCBI accession NP_001119584.1) and the cDNA of Cos-7 cells (NCBI accession NP_001040616.1), respectively, and cloned into the *Hind*III-*Eco*RI sites of the pCR3 vector (Invitrogen, CA, USA) [27]. The pcDNA3-GFP plasmid from addgene (Catalog# 74165) was used as a co-transfection control. All plasmids were Sanger sequenced to confirm the presence and accuracy of the cloned genes.

### Cell lines and *in vitro* co-transfection

C33A is an HPV-negative human cervical cancer cell line with an elevated expression of p53 R273C [53,54]. H1299 is a human lung squamous cell carcinoma cell line lacking expression of the p53 protein. Both cell lines were purchased from ATCC (Manassas, VA, USA). Vero cells isolated from the kidney of an African green monkey which stably expresses the wild-type p53 was a gift from Prof. Xiaofeng Guo (South China Agricultural University). Cell lines were maintained in Dulbecco’s modified Eagle’s medium (DMEM) (Gibco, MA, USA) with 10% fetal bovine serum (FBS) (Gibco, MA, USA). Cells at 60–70% confluence were co-transfected with E6 and p53 plasmids using Lipofectamine 3000 (Invitrogen, CA, USA) according to the manufacturer’s protocol, and then cultured for 24–48 hours at 37°C. To inhibit the proteasome function of E6-mediated p53 degradation, cells were treated with 10 μM MG132 20 hours after transfection, and then incubated for 4 hours before harvest.

### Western blot analysis

Cells were lysed in 2x sodium dodecyl sulphate (SDS) sample buffer to harvest the total protein as previously described [55]. Equal amounts of denatured protein lysates were separated by SDS-PAGE and western blot membranes were probed with antibodies recognizing the HA-tag (C29F4, Cell Signaling, MA, USA) and GFP (sc-9996, Santa Cruz, TX, USA). Blots were visualized using Clarity™ Western ECL Substrate (Bio-Rad, CA, USA) and images were captured using a ChemiDoc™ Imaging System (Bio-Rad, CA, USA).

### Double immunofluorescence assay

Optimized double immunofluorescence assays were performed as previously described [56]. In brief, C33A or Vero cells were seeded on coverslips in 6-well plates and transfected with 2 μg indicated E6 plasmids. After incubation for 24 hours at 37°C, cells were fixed with pre-cooled methanol and simultaneously probed with anti-mouse monoclonal p53 antibody (sc-126, Santa Cruz, TX, USA) and anti-rabbit monoclonal HA antibody (C29F4, Cell Signaling, MA, USA) at 4°C overnight. Cells were then incubated with a goat anti-mouse IgG antibody conjugated to Alexa Fluor 488 (Thermo Fisher, MA, USA) diluted 1/500 for 2 hours at 37°C, a goat anti-rabbit IgG antibody conjugated to Alexa Fluor 568 (Thermo Fisher, MA, USA) diluted 1/500 for 2 hours at 37°C, and lastly stained for nucleus with DAPI (Thermo Fisher, MA, USA). Coverslips were mounted onto slides and observed under Leica DM1000 LED (Leica Biosystems, Wetzlar, Germany).

### Site-directed mutagenesis

Site-directed mutagenesis was performed using the GeneArt Site-Directed Mutagenesis System (Invitrogen, CA, USA) according to the manufacturer’s instructions. Briefly, plasmid DNA was methylated and amplified in a mutagenesis reaction with two overlapping primers containing the target mutation. Recombination reaction *in vitro* was performed to circularize the PCR products. A final transformation of the circularized mutated DNA into DH5α-T1 digested the methylated parental DNA, leaving behind only the intact unmethylated mutagenesis reaction product. Information on the primers used for site-directed mutation is available upon request. All mutations were confirmed by Sanger sequencing of the complete E6 and p53 ORFs.

### Co-immunoprecipitation

Co-immunoprecipitation was performed by co-transfecting 3 μg of indicated HA-E6 plasmids and 3 μg of p53 plasmids in H1299 cells for 20 hours at 37°C. Cells were then treated with 10 μM MG132 for 4 hours and lysed using high-salt E1A buffer (500mM NaCl, 0.1% NP-40, and 50 mM HEPES [pH 7.0]) in the presence of a protease inhibitor. The cell lysates were filtered through syringe and centrifuged at 16,000g for 10 min. The supernatant was harvested and incubated with anti-rabbit monoclonal HA antibody (C29F4, Cell Signaling, MA, USA) in 1/300 overnight at 4°C. The mixture was incubated with thawed EZview™ Red Protein A beads (Sigma-Aldrich, MO, USA) for 2 hours at 4°C, followed by triple washes with 1x PBS containing 0.5% Triton X. The beads were subjected to western blot analysis.

### Modeling of the E6/E6AP/p53 complex structure

Structure of the E6/E6AP/p53 complex was predicted using the SWISS-MODEL based on the known amino acid sequences available in the Protein Data Bank (PDB) (PDB code: 4XR8), and then visualized using PyMOL [57]. The intra-molecular contacts were also indicated by PyMOL.

## Supporting information

S1 Table Amino acid mutations of the E6-binding domain (aa 94–292) of p53 between humans and macaques. (DOCX)

S1 Data List of papillomavirus genomes used in this study. (XLSX)

S1 Fig Estimated structures of macaque (A) and human (B) E6/E6AP/p53 complexes. Surface representation of p53, E6 and E6AP domains are colored in blue, tan and grey, respectively. Three indicated p53 residues (aa 104, 129 and 206) are denoted in red. Views of sub-interfaces of these three mutations are shown in the boxes on the bottom panel of the figure. Direct polar interactions are presented with dashed lines. Residues of p53 and E6 are indicated in upper- and lower-case, respectively.
(TIF)

S2 Fig Degradation of human and monkey p53 mutants mediated by HPV16 and MfPV3 E6s in C33A cells. Bar charts below the western blot images show the normalized expression levels of p53 against GFP. “MG132 +” and “MG132 –” indicate treatment with and without MG132, respectively.
(TIF)

S3 Fig Degradation of human and monkey p53 mutants mediated by HPV16 and MfPV3 E6s in Vero cells. Bar charts below the western blot images show the normalized expression levels of p53 against GFP. “MG132 +” and “MG132 –” indicate treatment with and without MG132, respectively.
(TIF)

S4 Fig Co-immunoprecipitation of HPV16 E6, human p53 and E6AP. Co-transfection of HA-E6 and Flag-p53 in H1299 cells were immunoprecipitated by HA-E6 and the proteins were immunoblotted for native p53 and E6AP.
(TIF)

S5 Fig Degradation of p53 proteins mediated by macaque PV E6s encoded by major NHP-PV species and genera. C33A and Vero cells were used for co-transfection of human and monkey p53 plasmids, respectively. “MG132 +” and “MG132 –” indicate treatment with and without MG132, respectively.
(TIF)
