# Supplementary material for: Non-human primate papillomavirus E6-mediated p53 degradation reveals ancient evolutionary adaptation of carcinogenic phenotype to host niche
Source: PLoS Pathog. 2022 Mar 25;18(3):e1010444. doi: 10.1371/journal.ppat.1010444 (PMC8986119; doi:10.1371/journal.ppat.1010444)
Supplement: S1 Table — (DOCX) [file ppat.1010444.s001.docx]

**S1 Table. Amino acid mutations of the E6-binding domain (aa 94 – 292) of p53 between humans and macaques.**

| AA site* | Human p53 | Monkey p53 |
| --- | --- | --- |
| **Q104H** | Hydrophilic; polar aa with uncharged R groups | Hydrophilic; Basic aa |
| **A129D** | Hydrophobic | Hydrophilic; Acidic aa |
| T155S | Hydrophilic; polar aa with uncharged R groups | Same with T |
| **L206S** | Hydrophobic | Hydrophilic; polar aa with uncharged R groups |
| L289F | Hydrophobic | Hydrophobic |

* The amino acid (AA) sites studied in this work are highlighted in bold.
