# Supplementary figures and images for: Non-human primate papillomavirus E6-mediated p53 degradation reveals ancient evolutionary adaptation of carcinogenic phenotype to host niche
Source: PLoS Pathog. 2022 Mar 25;18(3):e1010444. doi: 10.1371/journal.ppat.1010444 (PMC8986119; doi:10.1371/journal.ppat.1010444)

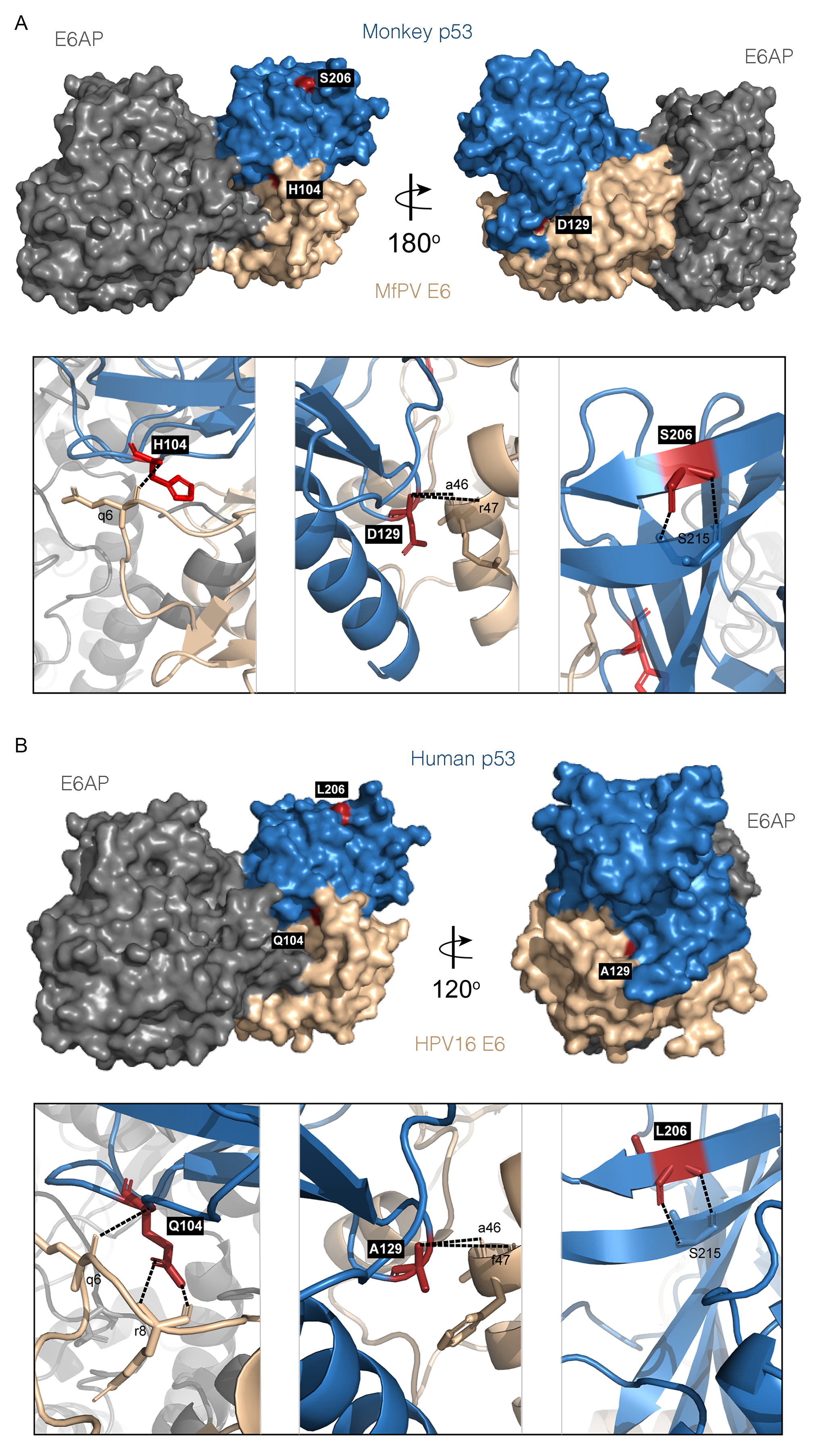

Supplement: S1 Fig — Surface representation of p53, E6 and E6AP domains are colored in blue, tan and grey, respectively. Three indicated p53 residues (aa 104, 129 and 206) are denoted in red. Views of sub-interfaces of these three mutations are shown in the boxes on the bottom panel of the figure. Direct polar interactions are presented with dashed lines. Residues of p53 and E6 are indicated in upper- and lower-case, respectively. (TIF) [file ppat.1010444.s003.tif]

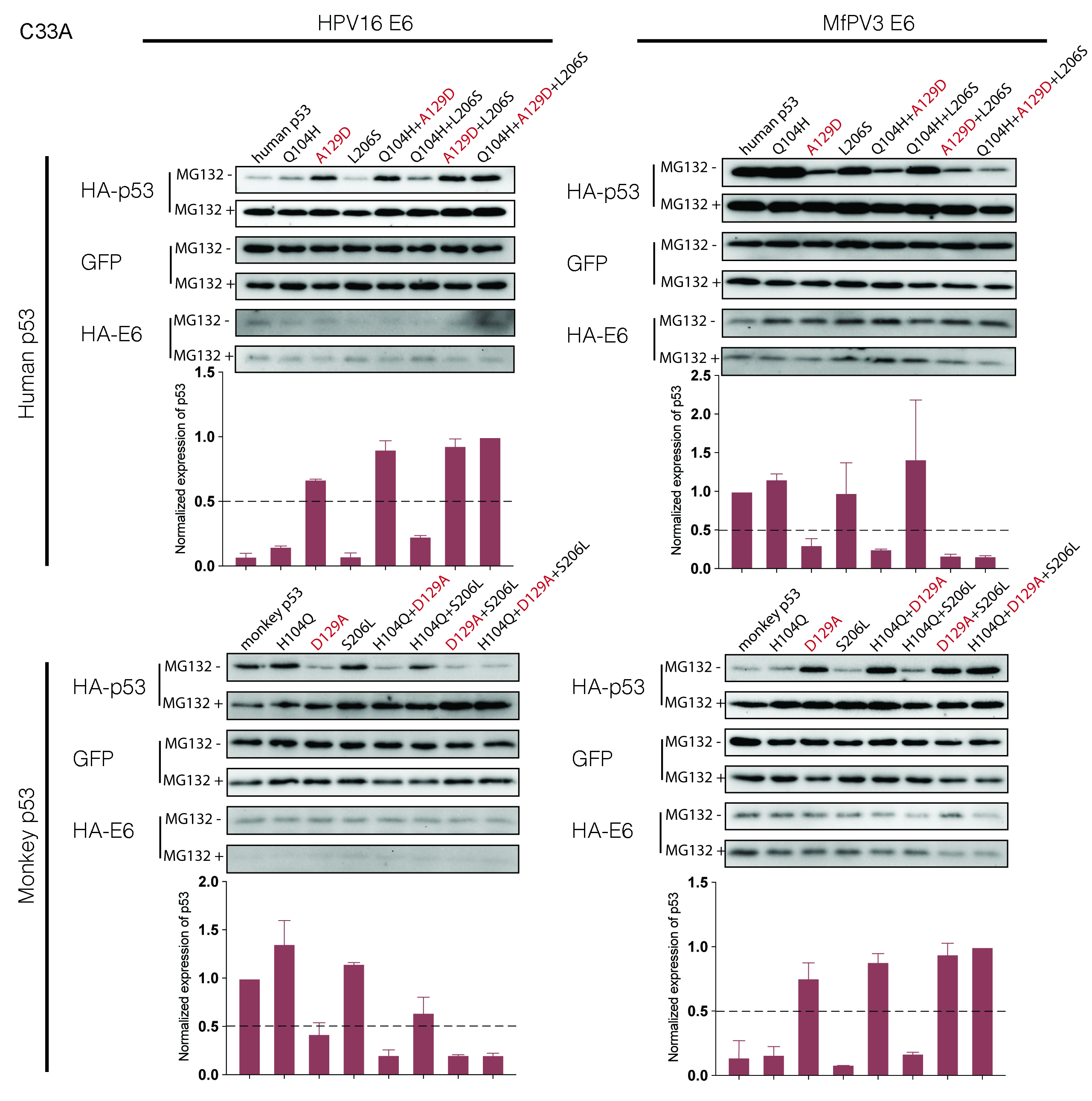

Supplement: S2 Fig — Bar charts below the western blot images show the normalized expression levels of p53 against GFP. “MG132 +” and “MG132 –” indicate treatment with and without MG132, respectively. (TIF) [file ppat.1010444.s004.tif]

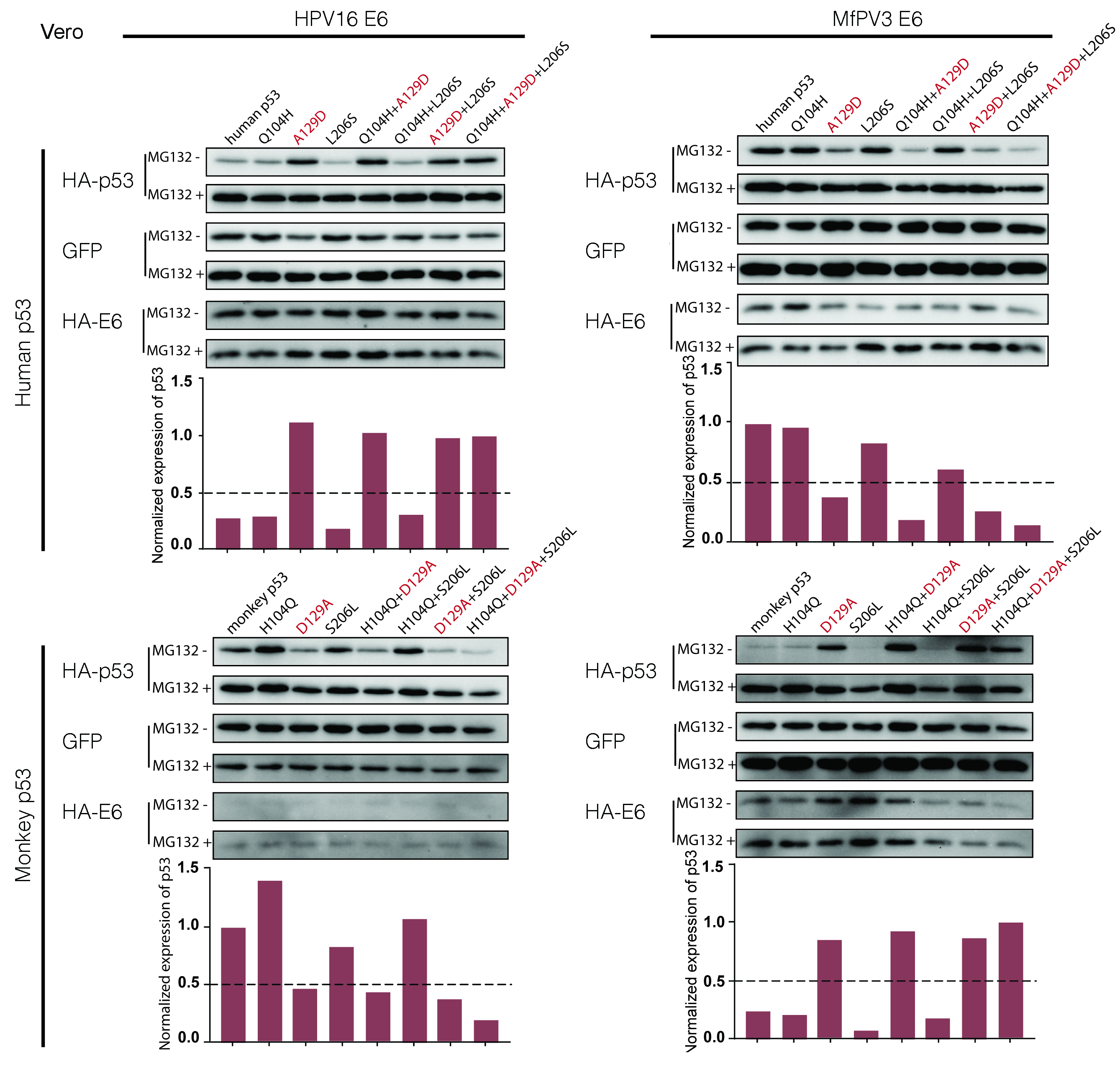

Supplement: S3 Fig — Bar charts below the western blot images show the normalized expression levels of p53 against GFP. “MG132 +” and “MG132 –” indicate treatment with and without MG132, respectively. (TIF) [file ppat.1010444.s005.tif]

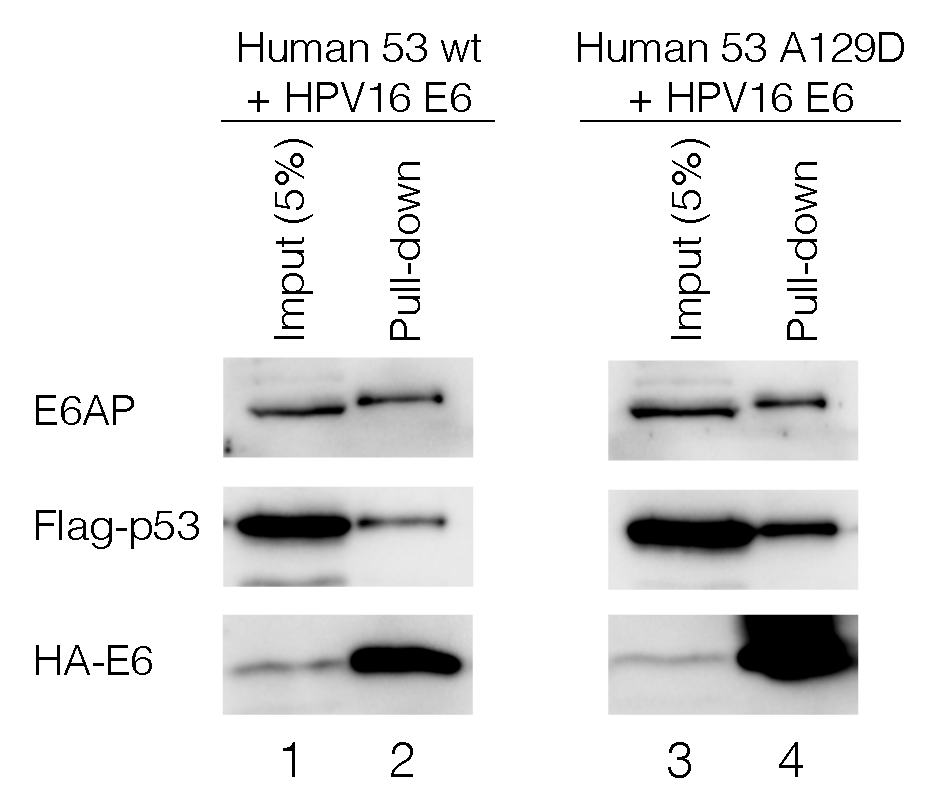

Supplement: S4 Fig — Co-transfection of HA-E6 and Flag-p53 in H1299 cells were immunoprecipitated by HA-E6 and the proteins were immunoblotted for native p53 and E6AP. (TIF) [file ppat.1010444.s006.tif]

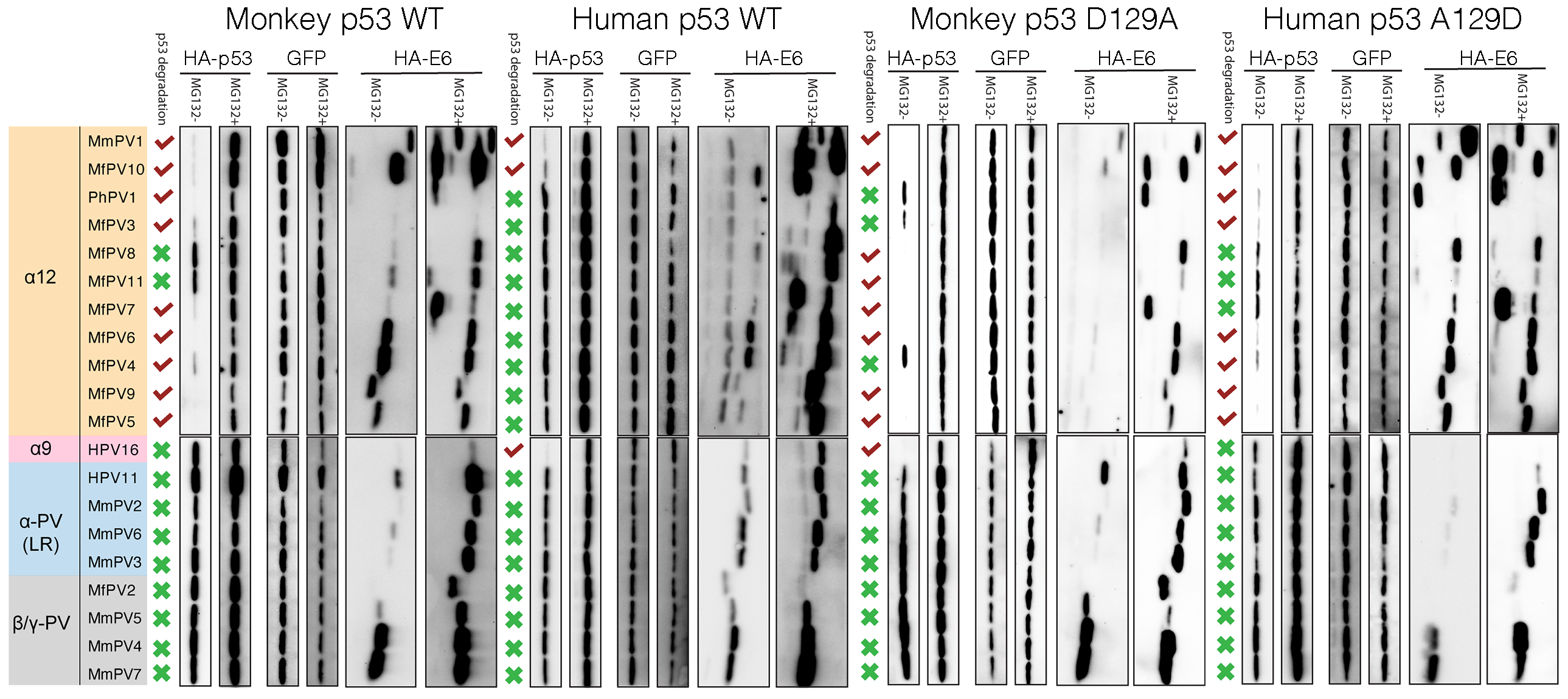

Supplement: S5 Fig — C33A and Vero cells were used for co-transfection of human and monkey p53 plasmids, respectively. “MG132 +” and “MG132 –” indicate treatment with and without MG132, respectively. (TIF) [file ppat.1010444.s007.tif]
